# Food-related behaviours among individuals with overweight/obesity and normal body weight

**DOI:** 10.1186/s12937-018-0401-7

**Published:** 2018-10-16

**Authors:** Anna Brytek-Matera, Kamila Czepczor-Bernat, Dominik Olejniczak

**Affiliations:** 1 Katowice Faculty of Psychology, SWPS University of Social Sciences and Humanities, Katowice, Poland; 2 Department of Public Health, Faculty of Health Science, Medical University of Warsaw, Warsaw, Poland

**Keywords:** Problematic eating behaviours, Emotional eating, Snacking, Chocolate, Body image, Body mass index

## Abstract

**Background:**

Emotional eating is a factor associated with a negative body image and other problematic eating behaviours. In this context little is known about differences between individuals with overweight and obesity and those with normal body weight. The main aim of the study was to evaluate the role of emotional eating in the relationship between the desire to consume chocolate and the wish to avoid social situations related to food and body exposures. For this purpose, we tested the direct, indirect and buffer effects. In addition, we used moderated mediation by introducing snacking into the model.

**Methods:**

The study included 123 outpatients with excessive body weight and 123 individuals with normal weight. The mean of body mass index (BMI) in the first group was 30.19 kg/m^2^ (*SD* = 4.37) and, in the second, it was 23.02 kg/m^2^ (*SD* = 1.20). The Three-Factor Eating Questionnaire, the Attitudes to Chocolate Questionnaire and the Body Image Avoidance Questionnaire were used.

**Results:**

Results show that in all individuals, the greater emotional eating is, the greater the desire for chocolate consumption and avoidance of social situations related to food and body exposures are. In addition, the desire for chocolate consumption are positively associated with avoidance of social situations related to food and body exposures in both group. Among individuals with excessive and normal body weight, emotional eating is a significant mediator in the relationship between desire for chocolate consumption and avoidance of social situations related to food and body exposures. However, it does not moderate the relationship between these variables. Outcomes show that there is a significant model of moderated mediation of the link between social situation–avoidance related to food and body exposure and the desire to consume chocolate through emotional eating, moderated by snacking among individuals with normal body weight. A similar effect has not been discovered in the group with excessive body weight.

**Conclusion:**

The presented results show that among people with varied BMI categories, emotional eating is connected to craving chocolate and avoidance of social situations related to food and body exposure that plays only the role of mediation. In addition, snacking is crucial for this relationship among the group with normal body weight.

## Background

The development of eating habits is a poly-aetiological process [1, 2]. Taking into account the presented research, the role of psychological factors seems to be of particular importance. This group includes cognitive [3, 4], familial [5], social [6, 7] and affective [8, 9] variables. The source literature argues that the majority of studies and publications relates to the relationship between emotional experiences and eating [10, 11].

The abnormal eating habits are divided into two categories [12–14]. The first is related to eating disorders (EDs), which are included in the Diagnostic and Statistical Manual of Mental Disorders-Fifth Edition (DSM-5) and the International Classification of Diseases (ICD-10) [15, 16]. In contrast, problematic eating behaviours (PEBs) are described in the literature as maladaptive eating habits (not included in either DSM-5 or ICD-10) [12, 17]. This category consists of unhealthy and pathological eating patterns, including emotional eating, snacking between meals and food cravings [17, 18] which are (in addition to the well-known role of eating disorders - especially binge eating disorder [BED]) crucial elements in the development of excessive body weight [12, 13].

The food might perform the role of a regulator of an emotional state [19, 20]. As the result of the experience of a specific valence affection, an individual can reduce negative emotions, increase the level of positive emotions or maintain the present emotional state through eating [21, 22]. In the literature on the relationship between emotions and food, there is a distinction between two types of hunger [1, 23]. What differentiates emotional hunger from physiological hunger is the moment when feelings of hunger, satisfaction, satiety and guilt appear [23]. The moment in which starvation, determined by emotions, occurs is sudden and associated with the desire to consume specific groups of products (*comfort food*). Food is taken to satiate the inner void, and its consumption continues despite the feeling of satiety. The last characteristic element of the incorrect eating process—related to emotional eating—is a greater likelihood of guilt arising after ingestion of food, as opposed to the situation when it is ingested to satisfy a biological need [23]. The emotional eating can be perceived as an abnormal eating habit that disturbs natural sensation of hunger and satiety and leads to body image concerns [17, 18].

The research on the relationship between eating and body highlights that experiencing one’s own body, especially body attitudes, is also related to the amount of consumed food [2, 10, 24, 25]. As a result of beliefs connected with the necessity to have a slim figure, the individual feels fear of gaining weight (especially in relation to the consumption of forbidden food, e.g., chocolate) and shame because of his or her own body, and acts to reduce these negative feelings [6]. The example of one such behaviour is the avoidance of social situations related to food and body exposure [26]. These situations are perceived as threatening (among others due to the possibility of losing control over the amount of food consumed in the presence of other people) and involve experiencing negative emotions, which in turn can promote the tendency to overeat under emotions [6, 26]. In addition, studies clearly show that overweight individuals have a stronger tendency to eat under the influence of experienced emotions than individuals with normal weight [27].

The behavioural aspect of the body image is related to such behaviours as weighing oneself and the use of various weight-loss methods [28]. These behaviours are manifestations of the absorption in body, which can cause stress and will be reduced by snacking [10, 29, 30]. The sweets/cake and fruit are the groups of products that most people eat between meals [31]. There is evidence in the literature that, in addition to emotional eating, snacking belongs to the group of variables crucial for the emergence and persistence of excess body weight [1, 10, 17, 18, 32].

The other approach of research on the eating process includes one focusing on the food cravings (problematic eating behaviour) in the context of chocolate consumption and their relationships to health [33]. The authors of the questionnaire measuring this relationship (together with emotional eating and guilt associated with chocolate consumption) specify the desire for chocolate consumption accompanied by inadaptable and persistent thoughts, beliefs and feelings [34, 35]. One of the symptoms of the desire to consume chocolate is the feeling that a person cannot stop thinking about the desire to eat chocolate [34]. In the context of mindful eating, individuals seeking to reduce their weight try to avoid chocolate (consider it as forbidden and a high-calorie product), and its consumption results in guilt and escalating dietary restrictions as well as a negative attitude toward one’s own body [29, 30].

It should be noted that little is known about relationship between abovementioned variables (emotional eating, body image and chocolate craving). On the basis of the literature [1, 10] we assumed that emotional eating was a factor associated with a negative body image and other problematic eating behaviours. From a clinical point of view, the more individuals, especially with problematic eating behaviours and disturbed body image-related behaviours (e.g. patients with excessive body weight) try to avoid thinking about food intake and their own body, the probability of eating a forbidden food (e.g. chocolate) and thinking about eating a forbidden food increase (which is based on the theory of paradoxical effects of thought suppression) [1]. Controlling thoughts are associated with stress and linked to increased tension, wherefore individuals try to reduce these negative states and may therefore be at risk of the over-consumption (e.g. emotional eating). Moreover, it is assumed that Frequent snacking leads to increased levels of stress (especially when individuals want to avoid eating and looking at their body) that may result in emotional eating and eating a forbidden food [1, 10, 17, 18, 32].

Numerous recent studies have analysed the mediating or/and moderating role of emotional eating on different psychological and nutrition variables, e.g. depression and weight gain [36], night eating with binge eating and body mass index [37]. Mediation analysis has become more meaningful in nutritional research and its use provides insight into the relationship among variables in a potential causal chain [38]. Therefore, the aim of this exploratory study was to (1) examine how the role of emotional eating in the relationship between desire for chocolate consumption and avoidance of social situations is related to food and exposure of the body (direct, indirect and buffer effect) and (2) whether the relationship between avoidance of social situations and desire for chocolate is mediated through emotional eating, moderated by snacking (the model of moderated mediation). On the basis of the studies [19, 27], it can be assumed that among individuals with excessive body weight, emotional eating will be a significant correlate, mediator and moderator of the relationship between variables related to desire for chocolate consumption and avoidance of body exposure.

## Methods

### Participants

Participants of this study included 246 individuals. All participants completed the self-reported weight and height. Exclusion criteria were BMI < 18.5 kg/m^2^ (underweight). A total of 123 outpatients with overweight and obesity (*M*_BMI_ = 30.19; *SD* = 4.37) and 123 participants with normal body weight (*M*_BMI_ = 23.02; *SD* = 1.20) participated in this study. At this point, it is worth noting that the indications from DSM-5 (278.00) describe overweight and obesity as conditions that may be a focus for clinical attention psychology and psychiatry (in opposition to category E66 in ICD-10, in which obesity is considered a problem of the endocrinologic, metabolic or nutritional aetiology) [15, 16].

The research (paper-and-pencil tests) lasted from January 2016 to March 2016 in various Polish institutions located in Silesia and Mazovia (including outpatients: treatment centres and participants with normal body weight: universities, companies). Compensation to research subjects was not offered. Demographic features are presented in Table 1.

**Table 1:** The demographic characteristic

	Excessive body weight BMI ≥ 25.00 kg/m^2^	Normal body weight 18.50 ≤ BMI ≤ 24.99 kg/m^2^
	*N* (%)	*N* (%)
Sex		
Female	78 (63.41)	87 (70.73)
Male	45 (36.59)	36 (29.27)
Education^a^		
Secondary education	44 (35.77)	40 (32.52)
Currently study yet	33 (26.83)	47 (38.21)
University education	39 (31.70)	34 (27.64)
	*M* (*SD*)	*M* (*SD*)
Age	32.18 (12.40)	26.05 (8.82)

*Note*. ^a^Seven indiviudals with excessive body weight and two individuals with normal body wieght did not complete the level of education

Additionally, in order to better understand participants functioning in the context of food-related and body-related behaviours, they were asked in sociodemographic survey about weighing (“Do you weigh yourself every day?”), methods of weight loss (“Are you currently taking action to reduce your weight?”) and snacking (“How often do you snack between meals”). More details about sociodemographic data are provided below (Table 2).

**Table 2:** Selected parts of the behavioural aspect of the body image among individuals with excessive and normal body weight

	Excessive body weight *N* (%)	Normal body weight *N* (%)
Every day weighing		
Yes	12 (9.76)	5 (4.07)
No	111 (90.24)	118 (95.93)
How often during the day?		
Once a day	13 (10.57)	9 (7.32)
Two times a day	2 (1.63)	1 (.81)
Three times a day	0	0
More than three times a day	0	2 (1.63)
Action to weight loss		
Yes	59 (47.97)	62 (50.41)
No	64 (52.03)	61 (49.59)
Methods of weight loss		
Dieting	25 (20.33)	19 (15.45)
Physical activity	35 (28.46)	46 (37.40)
Use of laxatives	1 (.81)	1 (.81)
Vomiting	1 (.81)	0
Starvation	1 (.81)	1 (.81)
Other	5 (4.07)	6 (4.88)
Snacking in between meals		
Always	2 (1.63)	8 (6.51)
Often	31 (25.20)	26 (21.14)
Sometimes	60 (48.78)	61 (49.59)
Rarely	27 (21.95)	26 (21.14)
Never	3 (2.44)	2 (1.63)
Products snacking in between meals (multiple choice)		
Fruits	51 (41.46)	46 (37.40)
Vegetables	4 (3.25)	1 (.81)
Sweets	20 (16.26)	59 (47.97)
Other	33 (26.83)	25 (20.33)

### Measures

Three questionnaires were used in the study:

1. The Three-Factor Eating Questionnaire (TFEQ-R18) [39], which contains 18 items that constitute three subscales: emotional eating (example: “When I feel blue, I often overeat”), uncontrolled eating (example: “Sometimes when I start eating, I just can’t seem to stop”), restrictive eating (example: “I consciously hold back at meals in order not to weight gain”). The study used the Polish version of the questionnaire [40]. Cronbach’s alpha reliability coefficient for “emotional eating” was .89 (excessive body weight: .92, normal body weight: .84).
2. The Attitudes Towards Chocolate Questionnaire [34, 35] includes 22 items that constitute three subscales connected with chocolate eating: guilt (example: “I feel guilty right after eating chocolate”), emotional eating (example: “I often eat chocolate when I am bored”), craving (example: “I like to indulge in chocolate”). Cronbach’s alpha reliability coefficient for the subscale “consumption desire” used in the study was .62 (excessive body weight: .63, normal body weight: .61).
3. Body Image Avoidance Questionnaire (BIAQ) [26, 41] consists of four subscales: clothing and appearance (example: “I wear baggy clothes”), physical appearance (example: “I wear clothes that will divert attention from my weight”), social activities (example: “I do not go out socially if the people I am with will discuss weight”), food and weight preoccupation (example: “I restrict the amount of food I eat”). Cronbach’s alpha coefficient for the subscale “social activity” used in the conducted studies (avoidance of social situations related to food and body exposure) was .90 (excessive body weight: .92, normal body weight: .86).

In the present exploratory study, firstly we used the full version of all questionnaires in order to do not affect and interfere with their reliability. Secondly, we solely used the subscales (for the analysis) related to the main aim of an a priori hypothesis.

### Data analysis

Statistical analysis of the collected data was performed by means of the Statistical Package for Social Sciences (version 22.0). The Pearson r correlation was used to verify the relationships among emotional eating, the desire to eat chocolate, and the avoidance of social situations related to food and body exposure. The PROCESS macro [42] with bootstrap *N* = 1000 was used to analyse the mediation and moderating effects and model of moderated mediation. All figures in the text were created on the basis of Hayes guidelines [42].

## Results

### Direct effect

The positive correlation between variables mentioned (emotional eating, chocolate craving and avoidance of social situations) are presented in Table 3.

**Table 3:** Emotional eating, the desire to consume chocolate as well as avoiding social situations related to food and body exposure among excessive and normal body weight individuals

	Excessive body weight	Normal body weight
Emotional eating ↔ Craving	.610^***^	.411^***^
Emotional eating ↔ Avoidance	.371^***^	.224^*^:::
Craving ↔ Avoidance	.282^**^	.153^*^

*Note*. ^*^ *p* < .05; ^**^ *p* < .01; ^***^ *p* < .001

### Indirect and buffer effect

**Fig. 1:**
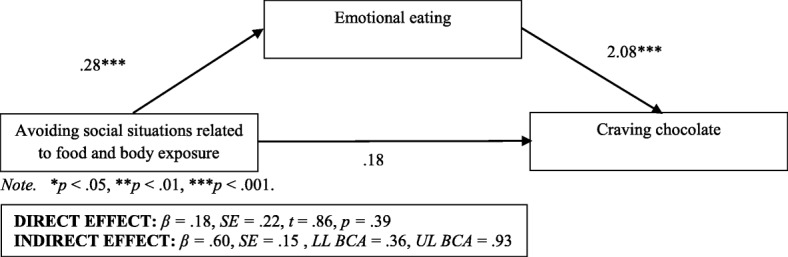
Emotional eating as a mediator in the relationship between avoiding social situations related to food and body exposure as well as the desire to consume chocolate among individuals with excessive body weight

Among all participants, emotional eating was a significant complete mediator of the relationship between social-situation avoidance related to food and body exposure and the desire to consume chocolate (Figs. 1 and. 2). Nevertheless, it did not moderate the relationship between these variables (Figs. 3 and 4, Table 4).

**Fig. 2:**
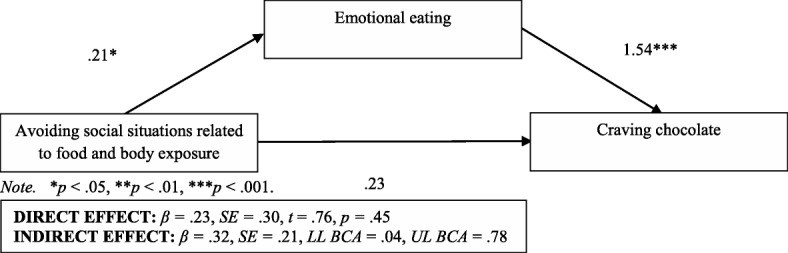
Emotional eating as a mediator in the relationship between avoiding social situations related to food and body exposure as well as the desire to consume chocolate among individuals with normal body weight

**Fig. 3:**
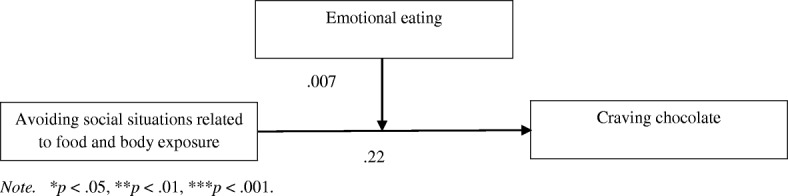
Buffer effect among individuals with excessive body weight

**Fig. 4:**
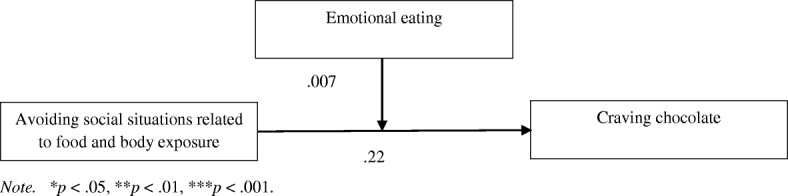
Buffer effect among individuals with normal body weight

**Table 4:** Emotional eating as a moderator in the relationship between avoiding social situations related to food and body exposure and the desire to consume chocolate among excessive and normal body weight individuals

	Effect	SE	t	LLCI	ULCI
Excessive body weight					
-1 *SD*	−.26	.30	−.83	−.87	.35
*M*	−.01	.22	−.06	−.44	.42
+ 1 *SD*	.23	.28	.81	−.33	.80
Normal body weight					
-1 *SD*	.19	.83	.23	−1.45	1.85
*M*	.22	.61	.36	−.98	1.42
+ 1 *SD*	.23	.78	.30	−1.32	1.78

### Moderated mediation models

**Fig. 5:**
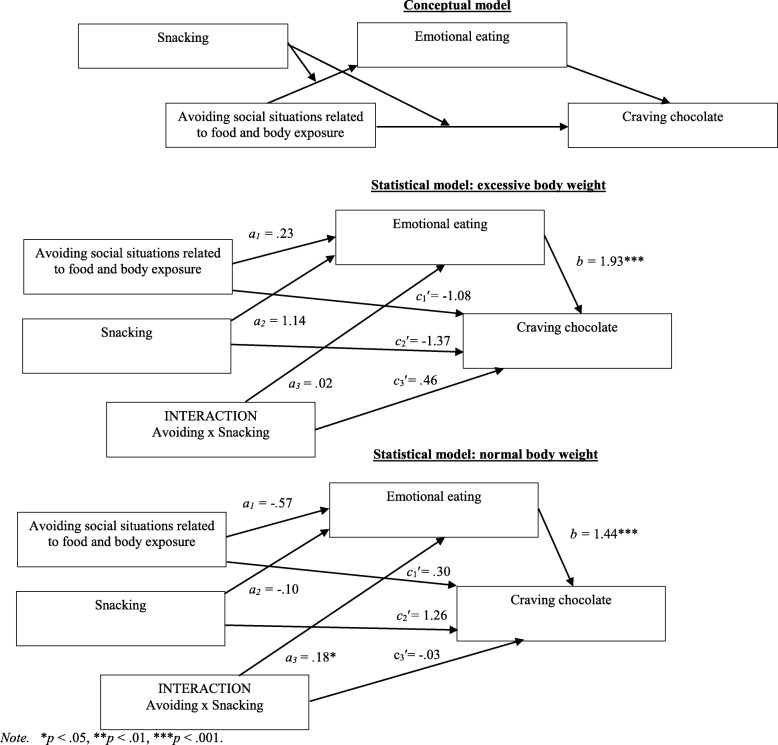
Conceptual and statistical model of moderated mediation the link between avoiding and craving through emotional eating, moderated by snacking

**Fig. 6:**
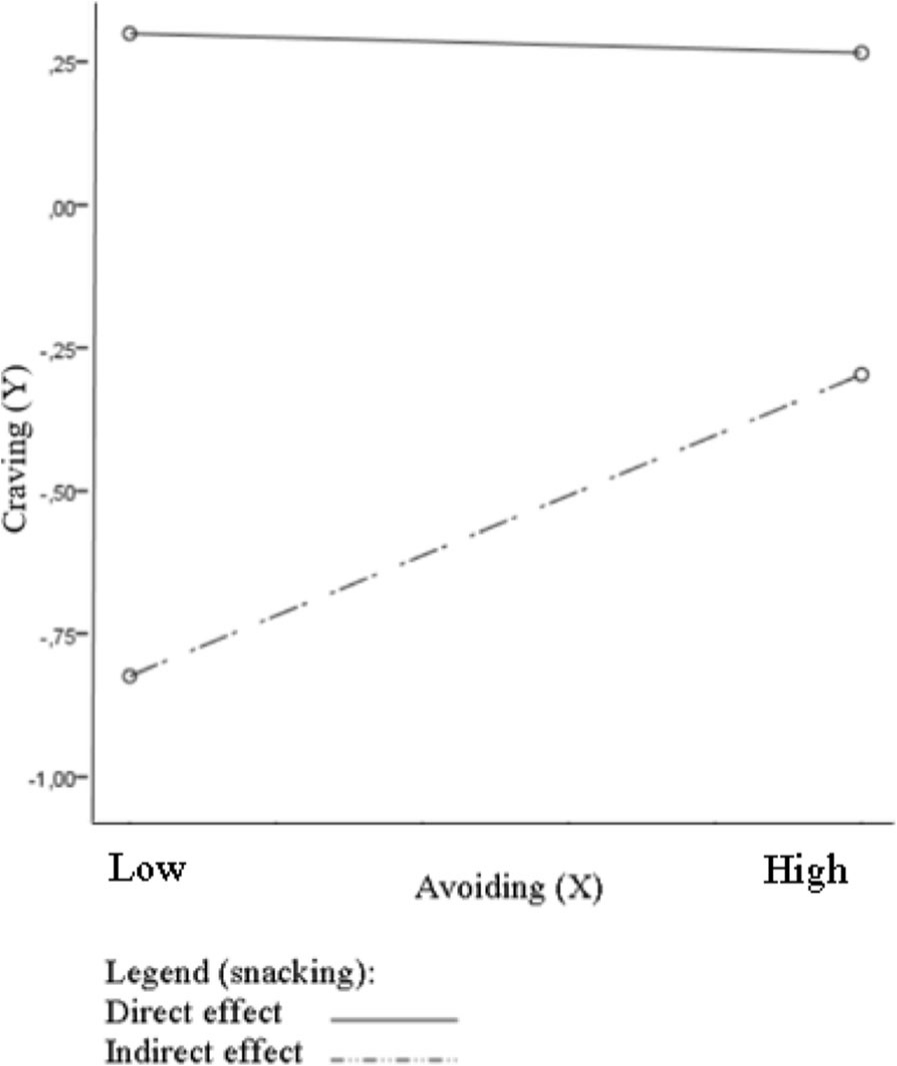
Moderating impact of snacking (W) on the mediating role of emotional eating (M) in effect of avoid social situations related to food and body exposure (X) on desire to chocolate (Y)

In relation to moderated mediation models, outcomes show that there was no significant model of moderated mediation of the link between social situation avoidance related to food and body exposure and the desire to consume chocolate through emotional eating, moderated by snacking among the group with excessive body weight, *R* = .51; *F*(3, 118) = 13.51; *p* < .001; *MSE* = 6.73; index of moderated mediation (for emotional eating as a mediator and snacking as a moderator): .05; [−.42, .58] (Fig. 5). Opposite results were obtained for this model of moderated mediation among individuals with normal body weight, *R* = .42; *F*(3, 119) = 8.69; *p* < .001; *MSE* = 5.13; index of moderated mediation: .26 [.05, .61] (Fig. 5). The conditional indirect effect of the social situation avoidance related to food and body exposure on desire to consume chocolate through emotional eating was significant in the high level of snacking (*B* = .19; [.004, .57] but was not significant in the low level of mediator (*B* = −.25; [−.70, .19]. In order to better understand the direction of the significant interaction, the simple slopes have been examined (Fig. 6).

## Discussion

In the study among all participants, the stronger the tendency to eat under the influence of emotions is, the greater the desire to consume chocolate and avoid social situations related to food and body exposure. In addition, the desire for chocolate consumption are positively associated with avoidance of social situations related to food and body exposures in both group. Moreover, in all groups, emotional eating is a complete mediator between avoiding food-related social situations and the need for body exposure as well as desire for chocolate consumption. A similar effect has not been discovered with regard to the buffer effect. To sum up, we failed to confirm the assumption that emotional eating will be a significant correlate and mediator only in the group with excessive body weight or its moderation role of emotional eating. In addition, results show that there is a significant model of moderated mediation of the relationship between social-situation avoidance related to food and body exposure and the desire to consume chocolate through emotional eating, moderated by snacking among individuals with normal body weight. It is worth pointing out that snacking is an important moderator of the relationship between social situation avoidance related to food and body exposure and emotional eating but not in the relationship between avoidance of social situations and desire to consume chocolate. Importantly, only at high level of snacking moderation takes place. A similar effect has not been discovered in the group with excessive body weight.

The results of the study did not confirm the particular importance of the tendency to eat under the influence of emotions for the manifested problematic eating behaviours and the attitude toward one’s own body only among individuals with overweight and obesity. Notwithstanding, such assumptions have already been presented in the classic monograph of Hilda Bruch [43], the author of the concept of emotional eating. According to Bruch [44] and contemporary researchers [23, 45, 46], the tendency to eat foods under the influence of emotion is due to the lack of adequate and accurate identification of signals of physiological hunger from feelings connected with the experienced emotions. The consequence of this eating style is overeating (to reduce negative feelings) and weight gain [45, 47]. Another theory is associated with the model of the mechanism of a vicious cycle of emotional regulation by eating, according to which negative emotions are the source of excitement that is mistakenly identified as a feeling of hunger. It contributes to the consumption of food, which in turn leads to a temporary reduction of negative emotions. However, this effect is short-lived; the level of negative emotions increases again, which promotes further consumption of food and weight gain [1, 19, 20].

The supplementation of our outcomes in Table 2 shows that there is a similar body attitude among overweight individuals and those of normal body weight. Both groups similarly control body weight, strive for a weight change and eat between meals. This shows that both groups at similar levels are bound up in their own body and control of the food they eat. This dependency may lead to the exhaustion of cognitive sources as a result of permanent control of body weight and the need for food monitoring among people seeking weight reduction (regardless of whether the current body weight is excessive or normal) [48, 49]. People are dissatisfied with their body and body mass as a result of the internalisation of the sociocultural ideal of a slim silhouette [6, 50]. They use large numbers of food restrictions (related to intake monitoring) which, consequently, leads to the loss of resources responsible for controlling the eating process and excessive binge eating (especially forbidden products: sweets, salty snacks and other high-calorie products) in a situation of a negative affect [49].

The results may also have the following explanations. (1) Individuals are at different stages of weight loss and, therefore, some people with normal body weight (who were previously obese), despite reduction of weight to the level described as normal, still continue to have abnormal dietary behaviours and use various methods of weight loss (the group with excessive body weight is at the beginning of weight reduction, and their eating habits are also abnormal) [51, 52]. (2) In both groups, there are people who use body mass reduction methods that are pathological (related to eating disorders) and serve to maintain normal body weight, despite the occurrences of binge eating (related to emotional eating and snacking) [53]. (3) The current fashion for a healthy lifestyle states that people are losing touch with their own needs and emotions (consequently, later confusing physiological and emotional needs), and strive to change their silhouette to the more perfect one [6, 46, 54]. (4) In the group of people with normal body weight, snacking between meals (besides emotional eating) is an important part of maladaptive eating and weight behaviours, and among people with excessive body weight, other crucial variables for abnormal eating patterns can be revealed (e.g., external eating, mindless eating) [18, 29, 30].

It is worth stressing that among people with excessive body mass, a high percentage (from 10 to 47.6%) are individuals with binge eating disorder (BED) and with bulimia [15, 55–57], but as mentioned earlier, eating disorders are also common in individuals with normal weight [6, 53]. There are many theories that emphasise the importance of emotions in eating disorders and persistence of excessive weight (including Emotional Reward Theory, Emotion Avoidance Model) [58, 59]. One of them is the integrative cognitive-affective therapy (ICAT) [60], which assumes that emotional dysregulation constitutes an important factor in the development of eating disorders and overweight. Low awareness of an emotional state, lack of flexibility in adaptive regulations, and a low level of control skills in stressful states contribute to binge eating that functions as a regulator of experienced emotions [60]. The analysis of the level of negative and positive emotions among individuals with excessive body weight and simultaneously indulging in BED has proved that the level of negative emotions significantly increased before binge eating and decreased afterward [61]. After some time, however, it increases again. The reverse trend has been observed in the case of positive emotions; before binge eating, such emotions decline, increase after the attack and then decline again. This indicates the importance of emotions of different valences for the development of abnormal habits and eating behaviours [61].

Other research suggests that body weight is a crucial aspect in the relationship between the tendency to eat under the influence of emotions and a body mass index [48]; only among overweight individuals is there a positive correlation between emotional eating and BMI. (This relationship is not relevant among participants with normal body weight.) The Homeostatic Theory of Obesity [24] also delineates the relationship between abnormal eating habits and negative emotions as well as negative body attitudes among individuals with excessive body weight. Its author [24], on the basis of a meta-analysis of studies on factors involved in the formation and persistence of obesity, emphasises that excessive body mass is associated with high levels of negative emotions, strong body dissatisfaction, and a frequent intake of high-calorie foods. In addition, these variables also interact with each other; high intensity of negative emotions contributes to the increased consumption of high-energy products, which in turn leads to higher body dissatisfaction. This theory is the confirmation of the assumption (which our outcome did not confirm) that food can act as a regulator of an emotional state, especially among overweight participants [24]. However, other authors [62] point out that this theory suffers from shortcomings and requires empirical development and verification, inter alia, because it does not take into account the motivational orientation (autonomous and controlled) that is crucial for the management of one’s own emotions, eating behaviours and body image [63–65].

Therefore, according to previous research results obtained by Micanti et al. [10] and Annesi et al. [66], snacking and emotional eating might be crucial for interventions related to changing eating behaviours, but not only for people with excessive weight. By addressing these problematic eating behaviours, future research should include longitudinal research to help recognise better mechanisms to change eating behaviours among individuals with various BMI levels (which may be in different stages of behavior change for weight loss, diet and exercise).

### Limitations

Our research has a few limitations. First, the acceptable level of an internal consistency for measures, using the Cronbach’s alpha, is .80 or higher [67]. In the present study, scale “consumption desire” falls below this cut-off. It should be noted that our results may be due either to the true relationships between variables (or lack thereof), or to the poor reliability of measures in the sample. Second, the number of women and men in the group is not the same. We have to take account of the fact that we should ensure a representative distribution of the population for this group to be considered a representative of groups of people to whom results will be generalised in the next research. In addition, individuals with excessive and normal body weight are not equal in other demographic characteristics (e.g. age). So, it should be highlighted that it can be influence on results. Moreover, additional information about the participants is lacking, such as history of physical/mental diseases, medication use, the weight change in the last year/months and it is known from other studies that these variables may have an impact on the eating behaviours and body-related behaviours [68–70]. Furthermore, the limitations are associated also with the kind of data, which was self-reported data. This type of data is burdened with the problem of self-presentation of the respondents and lack of possibility of objective verification. Finally, although snacking (based on a single item) has been shown to be predictive of related behaviours in our previous research [71] it should be emphasized that in the present study we used the subscale that have not been validated in Polish population.

## Conclusion

The presented results show that among people with varied BMI categories, emotional eating is connected with craving chocolate and avoidance of social situations related to food and body exposure, which play only the role of mediation. In addition, snacking is crucial for this relationship in the group with normal body weight.
